# Pharmacological and Genetic Modulation of REV-ERB Activity and Expression Affects Orexigenic Gene Expression

**DOI:** 10.1371/journal.pone.0151014

**Published:** 2016-03-10

**Authors:** Ariadna Amador, Yongjun Wang, Subhashis Banerjee, Theodore M. Kameneka, Laura A. Solt, Thomas P. Burris

**Affiliations:** 1 Department of Molecular Therapeutics, The Scripps Research Institute, Jupiter, Florida, United States of America; 2 Department of Pharmacology and Physiology, Saint Louis University, Saint Louis, Missouri, United States of America; McGill University, CANADA

## Abstract

The nuclear receptors REV-ERBα and REV-ERBβ are transcription factors that play pivotal roles in the regulation of the circadian rhythm and various metabolic processes. The circadian rhythm is an endogenous mechanism, which generates entrainable biological changes that follow a 24-hour period. It regulates a number of physiological processes, including sleep/wakeful cycles and feeding behaviors. We recently demonstrated that REV-ERB-specific small molecules affect sleep and anxiety. The orexinergic system also plays a significant role in mammalian physiology and behavior, including the regulation of sleep and food intake. Importantly, orexin genes are expressed in a circadian manner. Given these overlaps in function and circadian expression, we wanted to determine whether the REV-ERBs might regulate orexin. We found that acute *in vivo* modulation of REV-ERB activity, with the REV-ERB-specific synthetic ligand SR9009, affects the circadian expression of orexinergic genes in mice. Long term dosing with SR9009 also suppresses orexinergic gene expression in mice. Finally, REV-ERBβ-deficient mice present with increased orexinergic transcripts. These data suggest that the REV-ERBs may be involved in the repression of orexinergic gene expression.

## Introduction

The circadian rhythm is an autonomous, 24-hour, self-sustained oscillation regulating the physiology and behavior of organisms ranging from bacteria to humans [1–7]. The master clock is located in the suprachiasmatic nucleus (SCN) in the hypothalamus and is maintained by a negative feedback loop in which a CLOCK-BMAL1 complex activates E-box containing genes, including *Cryptochrome* (*Cry1* and *Cry2*), and *Period* (*Per1*, *Per2*, and *Per3*). In turn, the CRY-PER complex inhibits the activating action of the CLOCK-BMAL1 heterodimers. The REV-ERBs have been demonstrated to be an essential part of the accessory loop by inhibiting the transcription of the core cock genes *BMAL1*, *NPAS2*, and *CLOCK*. [8–13]. The molecular oscillations that occur in the brain also occur in peripheral organs and are involved in the regulation of metabolic functions [12, 14–16].

Recent data from our laboratory have demonstrated that the REV-ERBs are also involved in the maintenance of sleep architecture and anxiety [17, 18]. Using SR9009, a REV-ERB-specific synthetic agonist, we demonstrated that acute intraperitoneal injections at Zeitgeber time (ZT) 6 induced wakefulness during the subjective night period while chronic agonist administration reduced anxiety-like behaviors in mice. Conversely, mice deficient in REV-ERBβ displayed enhanced anxiety in different behavioral paradigms [19]. In a separate study, Jager *et al* demonstrated that mice lacking *REV-ERBα* displayed a hyperactive phenotype and decreased habituation in novel object paradigms as well as impaired short- and long-term memory. This behavior was determined to be due to direct regulation of tyrosine hydroxylase by REV-ERBα [20].

Extensive work has highlighted the importance of the lateral hypothalamic area (LHA) in wakefulness, feeding behavior, and energy metabolism [21, 22]. Prepro-orexin [PPO, (*Hcrt*)], also known as hypocretin, is a precursor neuropeptide, which generates orexin-A (hypocretin-1) and orexin-B (hypocretin-2). Orexin-A and -B peptides are exclusively produced in the lateral (perifornical region) and dorsal hypothalamus [23–28] and bind the G-protein coupled orexin 1 and 2 receptors, (OX_1_R and OX_2_R), respectively, also known as Hypocretin-1 Receptor (*HCRTR1*) and Hypocretin-2 Receptor (*HCRTR2*). The orexin peptides have been shown to play key roles in sleep, energy metabolism and feeding [22, 29–32]. In the brain, orexin signaling, particularly via OX_1_R, is involved in reward behavior, related to feeding, and drugs of abuse, while orexinergic signaling via OX_2_R is classically involved in wake maintenance [23, 33–39]. *Hcrt* has been previously shown to display a circadian pattern of expression during a 24h cycle in the hypothalamus [40]. Hypothalamic orexin neurons receive and extend projections to and receive projections from various regions in the brain, including the SCN [41]. The orexinergic neurons extending from the hypothalamus also innervate wake centers in the brainstem, including the locus coeruleus and the raphe nuclei, centers for sleep/wake regulation [25], and the peripheral autonomic nervous system [27]. Strikingly, mice lacking orexin are narcoleptic and develop late-onset obesity related to decreased energy expenditure [24, 42], supporting a key role for orexin in sleep-wake cycles and control of metabolic activity. Interestingly, circadian disruptions occurring due to mutations in core clock components, including the *Clock* gene, alter day-night expression levels of hypothalamic peptides including orexin [40], suggesting a link between orexin and the master clock. Moreover, orexin expression has been demonstrated to be expressed in a circadian manner [40]. Finally, the orexin pathway is thought to be involved in psychiatric disorders, such as anxiety, panic, depression, and schizophrenia, although very little is still known about its role [43–50].

Given the correlative links between the orexinergic and REV-ERB systems, specifically the regulation of circadian behaviors and metabolism, we investigated whether these two signaling pathways may converge and interact to regulate physiological processes. We demonstrate that REV-ERBs influence the transcription of the orexinergic genes in the hypothalamus and other centers in the brain. Given the intrinsic negative side effects associated with excessive motivation for food and lack of sleep, including anxiety, obesity, diabetes, and low self-esteem, our experiments suggest that the REV-ERB regulation of the orexin pathway may hold utility in ameliorating the detrimental effects of imbalanced circadian behavior via orexinergic pathways.

## Materials and Methods

### Mice

Male C57BL/6 mice, 8–10 weeks old, were obtained from Jackson laboratories (Bar Harbor, ME). *REV-ERBβ* KO mice were generated by breeding *REV-ERBβ* floxed mice (*Nr1d2*^*fl/fl*^) with *EIIa-Cre* mice obtained from Jackson Laboratories, Bar Harbor, ME.

Mice were housed under a standard 12h:12h Light:Dark cycle and fed *Ad libitum* with normal mouse chow (Harlan 2920X). Food was removed 4–5 hours prior to CO_2_ induced narcosis (method of euthanasia). All procedures were approved and conducted in accordance to the Scripps Florida Institutional Animal Care and Use Committee.

### Compound Administration

The formulation of REV-ERB agonists was performed as previously described [17]. SR9009 was formulated in a 10mg/ml solution of 15% cremophor, 85% purified water, and pH’d to 7.0. Injections of 100mg/kg, (volume: 10μl/gram) were performed intraperitoneally (i.p.) for all animal studies.

### General Mouse Studies

For acute injections, SR9009 was injected one time at either ZT0 (lights on) or ZT6, corresponding to the middle of the lights on period. ZT0 injections—groups of animals (n = 6) were sacrificed every six hours at ZT0, ZT6, ZT12 and ZT18. ZT6 injections- groups of animals (n = 6) were sacrificed one hour later at ZT7. For chronic studies, mice were administered SR9009 (100mg/kg, twice a day) for 10 consecutive days. Injections occurred at ZT0 (lights on) and ZT12 (lights off) in order to try and maintain exposure of SR9009 over a 24-hour period and be consistent with previously published dosing regimens of chronic SR9009 administration [17]. Animals (N = 5) were sacrificed at ZT6 after last injection. For REV-ERBβ KO mice, groups of animals (n = 5) were sacrificed at ZT6.

### RNA isolation and real time PCR

RNA was extracted from brain by homogenizing the tissue in 800 μL of RNA STAT-60, using a rotor tissue homogenizer (OMNI International). Chloroform was added after complete nucleoprotein complex dissociation at a 1:5 chloroform:STAT-60 ratio. Samples were vortexed for 15 minutes, kept at room temperature for 3 minutes and later centrifuged for 15min at 14,000rpm at 4°C. The supernatant was transferred to a new tube and half volume of isopropanol to that of STAT-60 was added. The samples were placed at room temperature for 5 to 10 minutes and centrifuged at 13,000 rpm, at 4°C for 10 min. The remaining pellet was washed twice with 75% ethanol, speed-vacuum-dried, and re-suspended in 20 μL of water. cDNA was synthesized using a qScript cDNA synthesis kit (Quanta BioSciences), using 1μg per sample in thermocyler (BioRad T100). Quantitative RT-PCR was performed using a 7900HT Fast RT-PCR (Applied Biosystems). Primers were designed using Primer3 (primer3.sourceforge.net). Specificity and validation of the primers were determined using an *In silico* PCR software program (genome.ucsd.edu) and melting curve analysis to eliminate the possibility of primer-dimer artifacts and check reaction specificity. Primer sequences for mouse REV-ERBα (*Nr1d1*), PPO (*Hcrt*), OX_1_R (*Hcrtr1*), OX_2_R (*Hcrtr2*), and Bmal1 (*Arntl*) genes are as follows:

*Nr1d1* forward:                     5’-ATGCCAATCATGCATCAGGT-3’

*Nr1d1* reverse:                      5’-CCCATTGCTGTTAGGTTGGT-3’

*Hcrt* forward:                        5’-AACCACGCTGCGGGTATCCT-3’

*Hcrt* reverse:                         5’-CCCTCCCCGGGGTGCTAAAG-3’

*Hcrtr1* forward:                     5’-GACTCTCAGCTTCATCGCCCT-3’

*Hcrtr1* reverse:                      5’-ACGCTGCTGCACTCCATGAC-3’

*Hcrtr2* forward:                     5’-GAGGATTCCCTCTCTCGTCG-3’

*Hcrtr2* reverse:                      5’-GGTGTAGGTATTCCCTCCACA-3’

*Arntl* forward:                       5’-CAGGCTAGCTTGATAGGACAGA-3’

*Arntl* reverse:                        5’-CCAGTGTAGGGGTGACTGTAAAC-3’

All data was normalized to Cyclophilin B (*Ppib*) based on stability and consistency of expression across all conditions analyzed:

*Ppib* forward:                       5’-GCAAGTTCCATCGTGTCATCAAG-3’

*Ppib* reverse:                        5’-CCATAGATGCTCTTTCCTCCTG-3’

### Statistical analysis

All data are expressed as mean ±S.E.M. All statistical analysis was performed using GraphPad Prism6 software. A two-way analysis of variance (ANOVA) was performed to determine significant differences between Time of day x treatment. One-way ANOVA was used to analyze intra-gene differences across several time points. All other analysis was performed using a Student’s *t*-test. (N is indicated in the figure legends). Significance was assessed as follows: * *p<0*.*05*, ** *p< 0*.*01*, ***p < 0.*001*.

## Results

**Fig 1:**
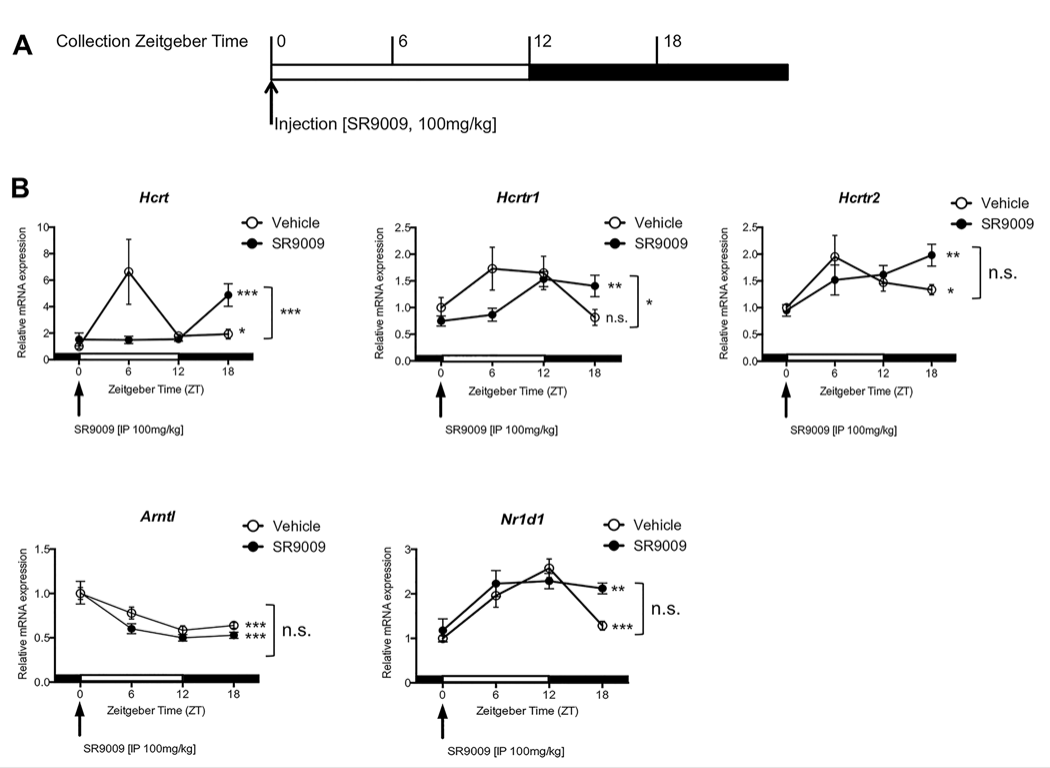
Administration of the REV-ERB agonist, SR9009, at ZT0 affects expression of orexinergic genes in the brain over a 24-hour period. **A.** Mice were injected at ZT0 with the REV-ERB agonist SR9009 [100mg/kg, i.p.] and tissue was collected every six hours for a twenty-four hour period. The brain was dissected into hypothalamus or reticular formation and processed for mRNA levels at the different time points, ZT0, ZT6, ZT12, and ZT18. **B.** Mice administered with SR9009 or vehicle control were assessed for expression of PPO (*Hcrt)*, Bmal1 (*Arntl*), and REV-ERBα (*Nr1d1)* in the hypothalamus and OX_1_R (*Hcrtr1*) and OX_2_R (*Hcrtr2*) in the brainstem. N = 6. One way analysis was used to determine intra-gene variation across time. Two-way ANOVA was used to assess differences between groups (time of day x treatment). *P<0.*05*, **P<0.*01*, and **P<0.*001*, *n*.*s*. *= not significant*.

In order to determine whether modulation of REV-ERB activity affected expression of PPO (*Hcrt*), OX_1_R (*Hcrtr1*), and OX_2_R (*Hcrtr2*), we administered SR9009 to mice at ZT0 and collected brain tissue every six hours to monitor gene expression changes (Fig 1A). Fig 1B shows that, consistent with published data, the mRNA expression of *Hcrt* fluctuates over a 24-hour period, peaking at ZT6 [40]. Surprisingly, SR9009 suppressed this peak in *Hcrt* expression at ZT6 relative to vehicle controls. Furthermore, SR9009 also affected the expression of *Hcrtr1* transcripts, inhibiting the initial upregulation of its expression at ZT6 relative to vehicle control. Similar effects were observed with SR9009 on *Hcrtr2*, although to a lesser degree. REV-ERBα contains a REV-ERB response element (RevRE) in its promoter region and has been shown to modulate its own expression. We also characterized the 24-hour expression pattern of the Bmal1 (*Arntl*) and REV-ERBα (*Nr1d1*) genes after ZT0 injections with SR9009 and observed repression of *Arntl* at ZT6 and little to no change in the expression of *Nr1d1* relative to vehicle control, which is consistent with our previously published data (Fig 1B) [17]. Thus, modulation of REV-ERB activity using a synthetic REV-ERB agonist affects expression of orexinergic genes over a 24-hour period.

**Fig 2:**
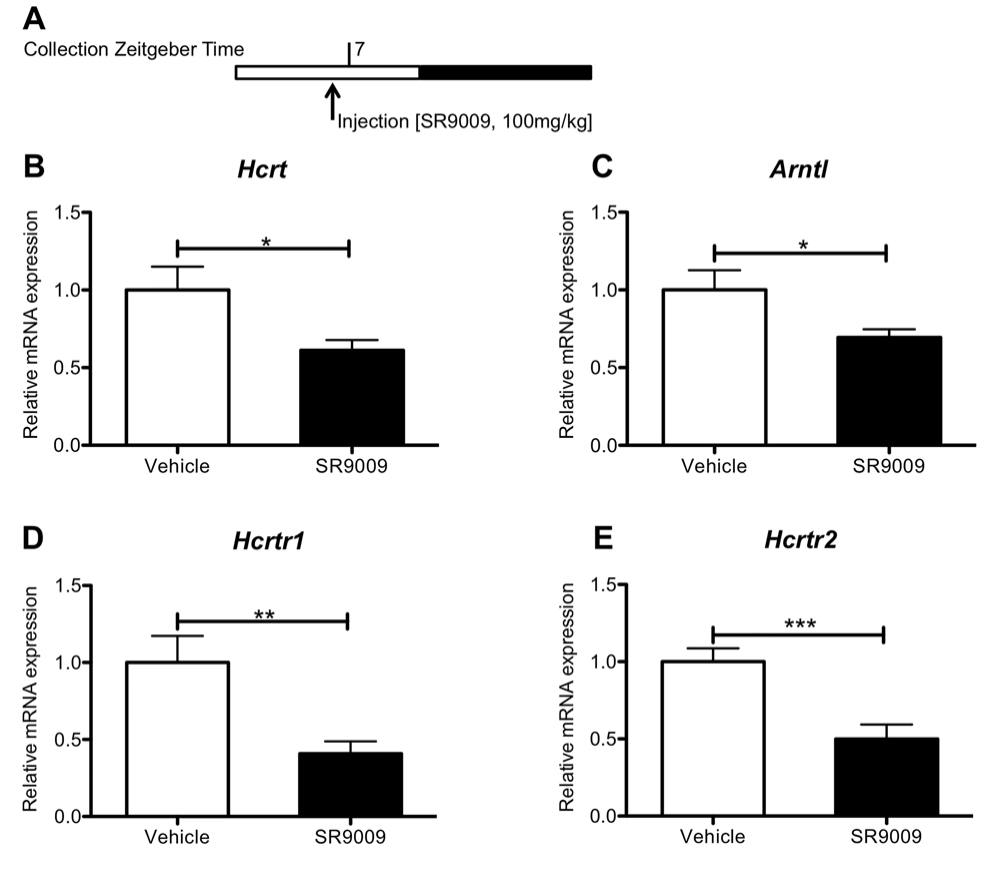
REV-ERB agonist SR9009 administration at ZT6 causes decreased transcript expression of orexinergic genes in the brain. **A.** Hypothalamic sections were analyzed for transcript levels of PPO (*Hcrt*) at ZT7. **B.** Hypothalamic sections were analyzed for transcript levels of Bmal1 (*Arntl*) at ZT7. **C.** Reticular formation sections were analyzed for transcript levels of OX_1_R (*Hcrtr1*) at ZT7. **D.** Reticular formation sections were analyzed for transcript levels of OX_2_R (*Hcrtr2*) at ZT7. N = 6. Student’s *t*-test was used to assess differences between groups. *P<0.*05*, **P<0.*01*, and ***P<0.*001*.

We recently demonstrated that acute injections at ZT6, which corresponds to peak REV-ERB mRNA expression, results in increased wakefulness and locomotion as well as reduced rapid-eye movement (REM) and slow-wave sleep (SWS) [51]. Since orexin is implicated in wake and alertness maintenance [33–36, 38, 52–58], we evaluated the effects of acute injections of SR9009 at ZT6 on orexinergic transcripts, as a possible mechanistic pathway mediating REV-ERB agonist wake-inducing effect. Mice were injected at ZT6 and sacrificed at ZT7, the time point where SR9009 has maximal wake-inducing effects, to collect brain tissue (Fig 2A). SR9009 suppressed *Hcrt* and Bmal1 (*Arntl*) in the hypothalamus (Fig 2B and 2C) and *Hcrtr1* and *Hcrtr2* transcript levels in the brainstem of mice (Fig 2D and 2E). *Arntl* was used as a positive control (Fig 2B).

**Fig 3:**
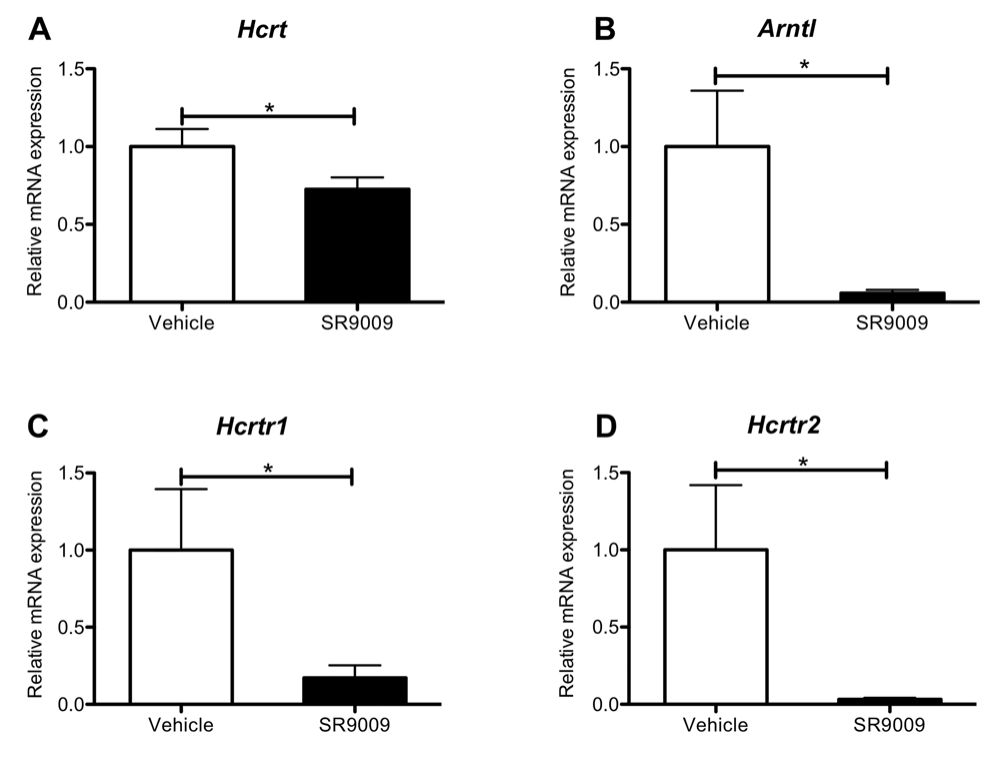
Chronic administration of SR9009 represses orexinergic transcription. Chronic administration of SR9009 results in decreased mRNA levels of **A.** PPO **(***Hcrt)* and **B.** Bmal1 (*Arntl*) in the hypothalamus at ZT6. Chronic administration of SR9009 results in decreased mRNA transcript levels of **C.** OX_1_R (*Hcrtr1)* and **D.** OX_2_R (*Hcrtr2)* in the brainstem at ZT6. N = 6. Student’s *t*-test was used to assess differences between groups. *P<0.*05*, **P<0.*01*, and ***P<0.*001*.

To determine how chronic administration of REV-ERB ligands affects expression of *Hcrtr1* and *Hcrtr2*, we administered SR9009 to mice for 10 days [100mg/kg, i.p., twice a day] after which time mice were sacrificed at ZT6 to collect brains for mRNA analysis. We assessed the expression of orexinergic genes in the hypothalamus, and the brainstem. In the hypothalamus, SR9009 inhibited *Hcrt* expression, with suppression of *Arntl* used as a control. (Fig 3A and 3B). Similarly, in the brainstem, mRNA expression of *Hcrtr1* and *Hcrtr2* were also reduced (Fig 3C and 3D respectively). These results indicate that the REV-ERBs may be repressing orexinergic transcription in these brain areas.

**Fig 4:**
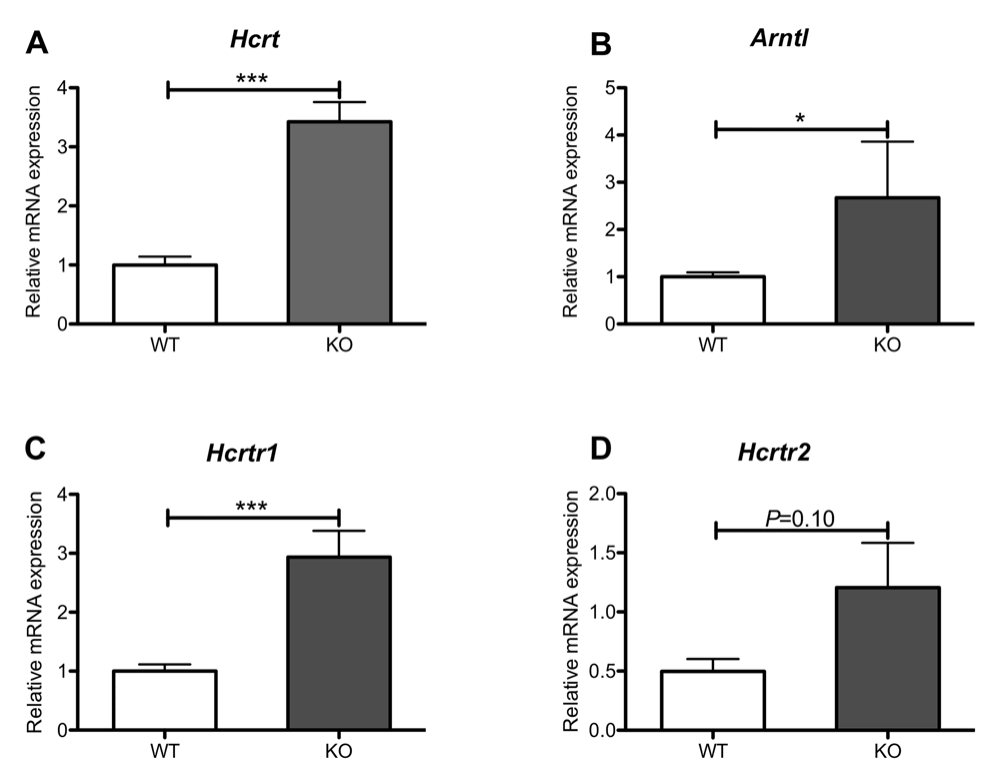
**Loss of REV-ERBβ leads to de-repression of orexinergic genes A**. PPO (*Hcrt)*, **B**. Bmal1 (*Arntl*) in the hypothalamus, and **C.** OX_1_R (*Hcrtr1)* **D.** and OX_2_R (*Hcrtr2)* transcript levels in the brainstem were assessed using RT-PCR. Increased orexinergic and *Bmal1* (*Arntl*) transcripts were observed at ZT6 in REV-ERBβ-deficient versus wild-type mice. N = 8. Student’s *t*-test was used to assess differences between groups. *P<0.*05*, **P<0.*01*, and ***P<0.*001*.

To determine how loss of REV-ERB expression affects orexin gene expression, we used full-body REV-ERB*β* deficient mice to assess *Hcrt* and *Arntl* levels in the hypothalamus, and *Hcrtr1* and *Hcrtr2* levels in the brainstem ([51] and upublished data). Consistent with REV-ERBβ being a transcriptional repressor, we observed increased expression of *Hcrt* and *Arntl* in the hypothalamus (Fig 4A and 4B) and of *Hcrtr1* and *Hcrtr2* in the brainstem (Fig 4C and 4D). These data suggest that REV-ERBβ may act as a transcriptional repressor of *Hcrt*, *Hcrtr1*, and *Hcrtr2*.

## Discussion

The REV-ERBs and orexin signaling regulate various aspects of food intake [23, 57, 59, 60], energy expenditure [23, 52, 58, 60–65], and sleep [33, 35, 36, 51, 53–56]. Orexinergic genes are expressed in a circadian manner [40] and the REV-ERBs have been shown to be critical regulators of the circadian rhythm [66]. Given the overlap in functions, we sought to determine whether the REV-ERBs may regulate orexinergic gene expression.

Our results show that modulation of REV-ERB activity affects transcription of the orexinergic genes [PPO (*Hcrt)*, OX_1_R (*Hcrtr1*), and OX_2_R (*Hcrtr2*)]. Acute pharmacological manipulation of REV-ERB activity resulted in alterations of orexinergic genes over a 24-hour period after single injections at ZT0, relative to vehicle control. Similar results were observed with chronic pharmacological manipulation of REV-ERB activity (Fig 3). Delayed/repressed transcription of the orexinergic genes suggests that they may be REV-ERB target genes (Fig 1). Alternatively, orexin is also regulated by peripheral nutrient signaling, directly affecting the hypothalamus [52, 58, 62–65] as well as the nucleus of the solitary tract (NTS) [67]. These signaling macronutrients and peptides may be activated by REV-ERB agonist administration, thus indirectly affecting orexinergic expression. Finally, modulation of orexigeneric gene expression may be due to post-translational effects at the genes, effects incited by nutrient signaling. Future studies examining this phenomenon are warranted in order to definitively determine whether this is the case.

Consistent with the REV-ERBs actively repressing orexinergic genes, mice deficient in REV-ERBβ demonstrate de-repression of orexinergic transcripts at ZT6, which would appear to further corroborate direct regulation of *Hcrt*, *Hcrtr1*, and *Hcrtr2* by the REV-ERBs. However, recently published ChIP-seq data generated by the Lazar laboratory determining REV-ERBα binding sites in the brain do not support this as no REV-ERBα binding to any regions in or surrounding orexigenic genes was observed [68]. However, in this data set, REV-ERBα does bind in the promoter region of the Nur77 gene (*Nr4a1*), along with other transcription factors that have been demonstrated to regulate orexin expression, which may account for the changes in orexinergic transcription observed in our studies [69]. REV-ERBα and REV-ERBβ are thought to bind to the same DNA response element and regulate similar genes [18, 70, 71]. In fact, REV-ERBβ is thought to be a redundant protein to REV-ERBβ [18, 71]. Therefore, the ChIP-seq data would suggest that the effects observed with SR9009 treatment and in the absence of REV-ERBβ are indirect effects. However, ChIP-seq studies determining the REV-ERBß cistrome in the brain, coupled with RNA-seq studies are warranted to definitively determine any direct/indirect effects of the REV-ERBs on orexin.

Orexin signaling via OX_1_R in the striatum and nucleus accumbens is associated with reward feeding and addictive behavior toward nicotine and other drugs [72–74]. In line with this, our lab recently demonstrated that administration of SR9009 injection decreased the addictive phenotypic properties of cocaine [19]. Suppression of reward behavior by the REV-ERBs may occur in part via orexinergic signaling. Future mechanistic studies may aid in describing the method of action of the REV-ERBs in reward. Therefore, pharmacological modulation of the REV-ERBs may be a viable therapeutic option to treat addiction, anxiety, and depression via regulation, at least in part, of the orexinergic pathway at the transcriptional level.

## Conclusions

Modulation of REV-ERB activity and expression leads to changes in expression of the orexinergic genes *Hcrt*, *Hcrtr1*, and *Hcrtr2*. Our laboratory recently published REV-ERB ligands effects on sleep, anxiety, and metabolism. Given the overlap in REV-ERB pathways with the orexinergic system, our data suggests there may be an interplay between the orexin and REV-ERB signaling pathways. However, further studies exploring this overlap are warranted. Collectively, our data indicate that REV-ERB ligands may be a means to regulate orexin expression and could be a potential therapeutic avenue for disorders associated with aberrant orexin signaling.
