# Correction: Pharmacological and Genetic Modulation of REV-ERB Activity and Expression Affects Orexigenic Gene Expression

**DOI:** 10.1371/journal.pone.0156367

**Published:** 2016-05-19

**Authors:** Ariadna Amador, Yongjun Wang, Subhashis Banerjee, Theodore M. Kamenecka, Laura A. Solt, Thomas P. Burris

The fourth author’s name is spelled incorrectly. The correct name is: Theodore M. Kamenecka. The correct citation is: Amador A, Wang Y, Banerjee S, Kamenecka TM, Solt LA, Burris TP (2016) Pharmacological and Genetic Modulation of REV-ERB Activity and Expression Affects Orexigenic Gene Expression. PLoS ONE 11(3): e0151014. 10.1371/journal.pone.0151014

## References

[1] Amador A, Wang Y, Banerjee S, Kameneka TM, Solt LA, Burris TP (2016) Pharmacological and Genetic Modulation of REV-ERB Activity and Expression Affects Orexigenic Gene Expression. PLoS ONE 11(3): e0151014 doi: 10.1371/journal.pone.0151014 26963516 10.1371/journal.pone.0151014 PMC4786293

